# The plasma virome in longitudinal samples from pregnant patients

**DOI:** 10.3389/fcimb.2023.1061230

**Published:** 2023-02-09

**Authors:** Molly J. Stout, Anoop K. Brar, Brandi N. Herter, Ananda Rankin, Kristine M. Wylie

**Affiliations:** ^1^ Department of Obstetrics and Gynecology, Division of Maternal Fetal Medicine, Washington University School of Medicine, St. Louis, MO, United States; ^2^ Department of Pediatrics, Division of Infectious Diseases, Washington University School of Medicine, St. Louis, MO, United States; ^3^ The McDonnell Genome Institute, Washington University School of Medicine, St. Louis, MO, United States

**Keywords:** virome, pregnancy, plasma, preterm birth, metagenomic sequencing

## Abstract

**Introduction:**

Nucleic acid from viruses is common in peripheral blood, even in asymptomatic individuals. How physiologic changes of pregnancy impact host-virus dynamics for acute, chronic, and latent viral infections is not well described. Previously we found higher viral diversity in the vagina during pregnancy associated with preterm birth (PTB) and Black race. We hypothesized that higher diversity and viral copy numbers in the plasma would show similar trends.

**Methods:**

To test this hypothesis, we evaluated longitudinally collected plasma samples from 23 pregnant patients (11 term and 12 preterm) using metagenomic sequencing with ViroCap enrichment to enhance virus detection. Sequence data were analyzed with the ViroMatch pipeline.

**Results:**

We detected nucleic acid from at least 1 virus in at least 1 sample from 87% (20/23) of the maternal subjects. The viruses represented 5 families: *Herpesviridae, Poxviridae, Papillomaviridae, Anelloviridae*, and *Flaviviridae.* We analyzed cord plasma from 18 of the babies from those patients and found nucleic acid from viruses in 33% of the samples (6/18) from 3 families: *Herpesviridae, Papillomaviridae*, and *Anelloviridae.* Some viral genomes were found in both maternal plasma and cord plasma from maternal-fetal pairs (e.g. cytomegalovirus, anellovirus). We found that Black race associated with higher viral richness (number of different viruses detected) in the maternal blood samples (P=0.003), consistent with our previous observations in vaginal samples. We did not detect associations between viral richness and PTB or the trimester of sampling. We then examined anelloviruses, a group of viruses that is ubiquitous and whose viral copy numbers fluctuate with immunological state. We tested anellovirus copy numbers in plasma from 63 pregnant patients sampled longitudinally using qPCR. Black race associated with higher anellovirus positivity (P<0.001) but not copy numbers (P=0.1). Anellovirus positivity and copy numbers were higher in the PTB group compared to the term group (P<0.01, P=0.003, respectively). Interestingly, these features did not occur at the time of delivery but appeared earlier in pregnancy, suggesting that although anelloviruses were biomarkers for PTB they were not triggering parturition.

**Discussion:**

These results emphasize the importance of longitudinal sampling and diverse cohorts in studies of virome dynamics during pregnancy.

## Introduction

Nucleic acid from DNA and RNA viruses, including members of the *Anelloviridae, Herpesviridae, Flaviridae*, and others, are common in peripheral blood (Popgeorgiev et al., 2013; Furuta et al., 2015; Rascovan et al., 2016; Moustafa et al., 2017). Many viruses in the blood have unclear pathogenicity, although some evidence of their association with clinical conditions has been recently reported (Kaczorowska and van der Hoek, 2020; Cebria-Mendoza et al., 2021a; Stapleton et al., 2011; Fama et al., 2020; Kyathanahalli et al., 2021). This evidence raises the possibility of vertical transmission of the highly prevalent blood viruses to the fetus during pregnancy (Racicot and Mor, 2017; Yu et al., 2022). In particular, the anelloviruses are one of the most abundant and diverse groups of viruses in healthy human blood (Cebria-Mendoza et al., 2021b), with some studies reporting >90% prevalence (Spandole et al., 2015). An inverse relationship between anellovirus genome copy numbers and immunocompetence is observed (Biagini, 2009; Dodi et al., 2021; De Vlaminck et al., 2013; Strassl et al., 2018; Frye et al., 2019), likely due to effects of immunological controls on viral replication. Increased prevalence of anellovirus genomes has also been observed in the plasma of children under 3 years old with unexplained fevers compared with controls (McElvania TeKippe et al., 2012). This could possibly be due to primary infection, since a role of anelloviruses in childhood respiratory diseases has been posited (Dodi et al., 2021), or due to proliferation of mononuclear cells, which are proposed to be the cells in which anelloviruses replicate (Maggi et al., 2001; Focosi et al., 2015). The mechanism(s) by which anelloviruses influence, and are influenced by, the host immune system is still emerging (Kaczorowska and van der Hoek, 2020).

We do not fully understand how the physiologic changes of pregnancy impact host-virus dynamics for acute, chronic, and latent viral infections. This is an important question to investigate, as many human viruses are present (often asymptomatically) in and on generally healthy, reproductive aged adults (Wylie et al., 2014), and therefore there is potential for subsequent effects on reproductive outcomes. Maternal viral infections can result in adverse events during pregnancy and poor fetal outcomes (Chudnovets et al., 2020; Yu et al., 2022). Pregnant patients are more susceptible to severe disease from some viral infections such as influenza, varicella, and COVID-19 when compared to the general population (Siston et al., 2010, Meijer et al., 2015, Silasi et al., 2015; Adhikari et al., 2022). Viral dynamics may also influence obstetric outcomes. For example, higher viral levels of hepatitis B viral load in cord blood are associated with an increased risk for preterm birth (PTB)(Elefsiniotis et al., 2011; Lu et al., 2014). In a systematic review and meta-analysis HIV infection was associated with a 50% increase for PTB, which was not attributable to adverse effects from antenatal antiviral HAART therapy (Wedi et al., 2016).These findings suggest that viral dynamics may reflect the underlying immune status of pregnant people and raises the possibility that viral communities may not cause overt symptoms, yet could be important markers of, or contributors to, obstetric disease.

In our studies of the vaginal virome in pregnant patients, we found that higher viral diversity is associated with PTB. We also found that higher viral diversity was associated with Black race, which is interesting as Black patients are also at higher risk for PTB compared to White patients (Xiao et al., 2015; Martin et al., 2022). The finding of racial differences in microbial communities in our work and others is not well explained. Mounting data suggests that observable biologic phenomenon, including poorer health outcomes, is linked to complex socio-cultural exposures including structural racism and chronic stress. Given our previous findings that characteristics of the vaginal virome are associated with both racial differences and an increased risk for PTB, we sought to examine the association between viral diversity in maternal plasma and PTB. Two previous studies have evaluated viruses in the plasma during pregnancy, but neither included race as a covariate nor evaluated longitudinally collected samples. In the first study, serum from patients with premature rupture of membranes and delivery before 37 weeks of gestation were compared with samples from patients delivering at term (Shah et al., 2020). Metagenomic sequencing was used to evaluate a subset of patients, followed by PCR assays for anelloviruses. The authors found that presence of nucleic acid from the anellovirus group TTV was associated with preterm premature rupture of membranes (PPROM). This study did not evaluate race as a covariate, analyze longitudinal samples, carry out quantitative PCR, or evaluate other types of PTB besides PPROM (Shah et al., 2020). A second study analyzed the virome using PCR assays on plasma and whole blood samples from a cohort of patients with preeclampsia, spontaneous preterm labor, or histologic chorioamnionitis (Sloan et al., 2022). They did not find an association between any components of the virome with any obstetric outcomes evaluated. This study also did not evaluate race as a covariate, evaluate samples collected longitudinally, or include term, healthy pregnancies for comparison. In the current study, we assessed viral nucleic acid in maternal plasma samples collected longitudinally throughout pregnancy (1st, 2nd, 3rd trimesters and at delivery), allowing us to evaluate dynamics of the virome throughout pregnancy from a racially diverse cohort. We used metagenomic sequencing along with ViroCap, an extremely sensitive viral genome enrichment panel we developed (Wylie et al., 2015), to obtain a broad view of the plasma virome in a subset of the samples, and then we carried out quantitative molecular assays for anelloviruses on samples from the full cohort to determine whether fluctuations in viral diversity and load associate with race or PTB.

## Materials and methods

### Subjects and samples

We performed a retrospective cohort study on stored plasma samples collected longitudinally from pregnant individuals with singleton pregnancies at routine prenatal care visits. Plasma was chosen instead of whole blood to measure nucleic acid from circulating viruses, rather than those that are latent or cell associated. To perform linked maternal and fetal analyses, subjects with samples were obtained in the 1^st^, 2^nd^, and 3^rd^ trimesters of pregnancy, at delivery, and had cord blood available were prioritized. A total of 23 patients were selected with a nearly complete set of maternal blood (during pregnancy and delivery blood), and a subset of 18 patients also had paired cord blood from the infant at delivery. This cohort was used for metagenomic sequencing with ViroCap. Subsequently an additional 40 subjects were added with maternal longitudinal (antepartum and delivery samples) to expand the sample size for anellovirus PCR assays. Samples were provided by the Women and Infants Health Specimen Consortium (WIHSC) Biobank at Washington University School of Medicine. Maternal blood was obtained through routine venipuncture, typically timed when clinical laboratories for obstetric care were obtained. Cord blood was obtained through venipuncture of the umbilical cord at the time of delivery. The Human Research Protection Office at Washington University School of Medicine approved this study (protocol number 201607029). Signed informed consent was obtained from all maternal patients enrolled in the study for the use of their specimens and for the use of cord plasma from their infants. Preterm birth was defined as birth at <37 weeks of gestation. Maternal race was self-reported.

### Nucleic acid sequencing and analysis

Total nucleic acid was extracted using the NucliSENS^®^ easyMAG^®^ Instrument (bioMerieux, Marcy l’Etoile, France). Dual-indexed sequencing libraries were constructed from total nucleic acid so both DNA and RNA viruses could be detected as previously described (Wylie et al., 2015; Wylie et al., 2018a). In brief, RNA in the total nucleic acid was reverse transcribed using reverse transcriptase (Promega) and random nonamers (Integrated DNA technologies). The random primers were tagged with a conserved sequence (5’-GTTTCCCAGTCACGATA-3’) that was used for subsequent amplification. Sequenase V2.0 DNA polymerase (Affymetrix) was used for second strand synthesis. DNA and RNA fragments were amplified with Accuprime Taq (Life Technologies) using primers targeting the conserved sequence from the random primers. The DNA/cDNA mixture was sheared with the Qsonica Q800R instrument (Qsonica) to generate ~500 bp fragments. Sequence libraries were constructed using the Accel NGS-2S library kit (Swift Biosciences, Ann Arbor, MI). Libraries were combined into two pools containing 42 or 43 human samples, and a mouse control sample was included in each pool. Viral sequences were enriched with ViroCap targeted sequence capture probes according to the manufacturer’s instructions (synthesized by Roche NimbleGen, Madison, WI). (Wylie et al., 2015) Sequences (2x150 bp)were generated on the Illumina HiSeq 1T instrument (Illumina, San Diego, CA). Sequences from human viruses were identified based on nucleotide and translated sequence alignment against reference genomes (Wylie et al., 2018a). Viral assignments were manually reviewed, and viral species and subtypes were confirmed. A background signal of 0.1% of the total signal from an individual virus in the pooled samples was subtracted to eliminate cross-sample contamination that occurs from pooling. (Wylie et al., 2015) Contamination was evaluated by looking for signal from human viruses in the mouse controls and signal from mouse viruses in the human samples. A low level of pegivirus was detected in the mouse sample in one pool, like the level found in other samples from that pool, and pegivirus was removed from the samples in that pool. No other contaminants were detected.

### Anellovirus quantitative PCR (qPCR)

Anellovirus genome copy numbers were quantified by real-time PCR with degenerate primers that target a highly conserved region of the untranslated region of the genome and detects all three genera of human anellovirus *(Alphatorquevirus*, *Betatorquevirus*, and *Gammatorquevirus*)(5’-ACWKMCGAATGGCTGAGTTT-3’ and 5’- CCCKWGCCCGARTTGCCCCT-3’) (Integrated DNA technologies) (Young et al., 2015). A 200 bp synthetic sequence containing the PCR target was synthesized (Integrated DNA Technologies, Inc., Coralville, Iowa) for use as a quantitative control. Assays were performed with the TB Green Advantage qPCR Prremix (Takara) on the ABI 7500 (Applied Biosystems). Samples in which anellovirus was undetected were set to the limit of detection (1000 copies per ml).

### Statistics

Data were plotted and compared using the R programming language (R Core Team, 2013), with the library Plotly (Sievert, 2019). Heatmaps were generated with the R library Pretty Heatmaps (Kolde, 2019). The libraries vegan and labdsv were used to calculate beta diversity, create non-metric multidimensional scaling plots, and calculate permutational multivariate analysis of variance using distance matrices (adonis) (Oksanen et al., 2019, Roberts, 2019). Dynamics over time were evaluated with generalized linear mixed effects models using the R library lme4 (Bates et al., 2015). Specifically, anellovirus positivity or viral load was the response variable; race, trimester, and preterm status were fixed variables; and subjects were included as random effects.

## Results

### Sequencing the plasma virome

**Figure 1:**
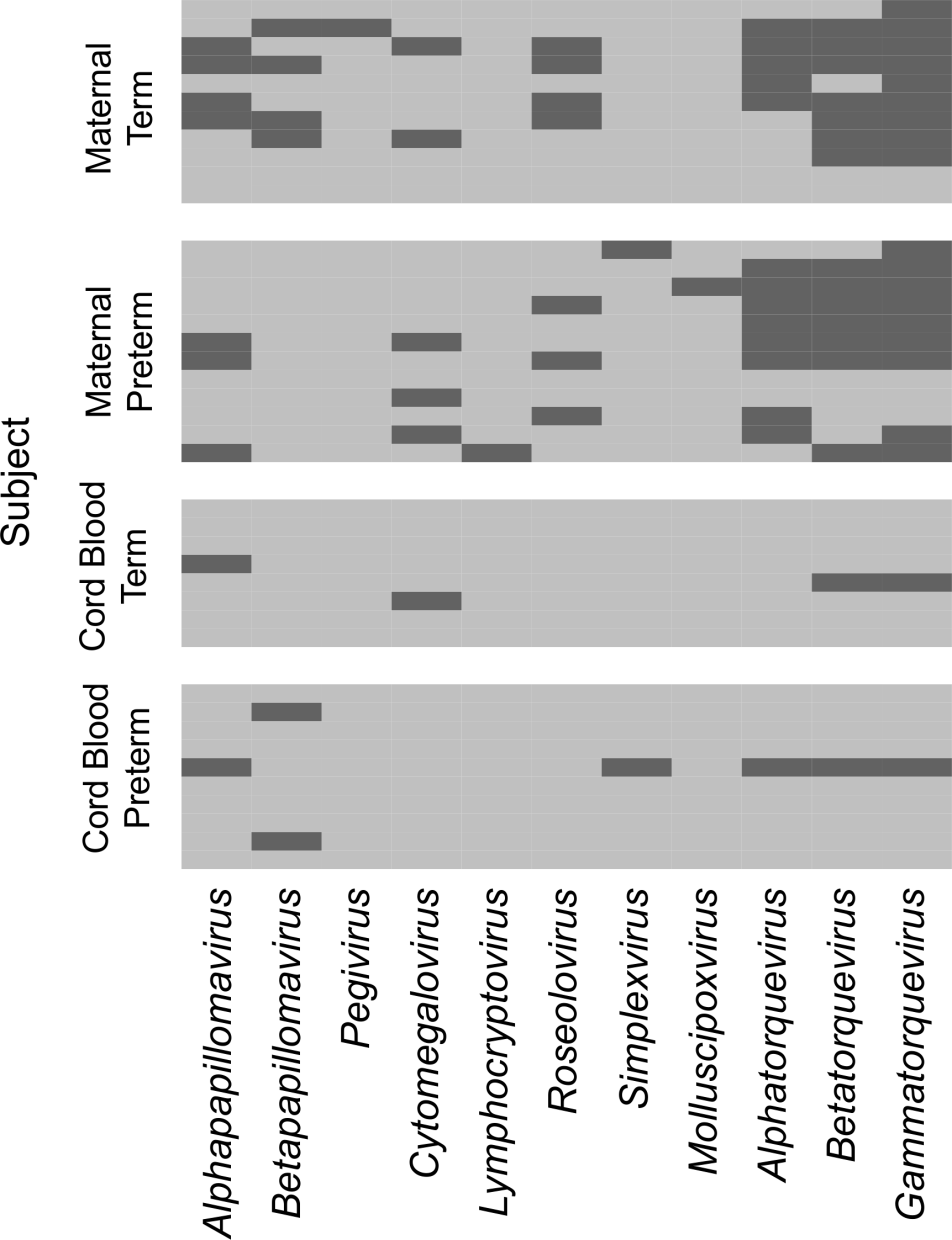
Viral genera detected in maternal and cord plasma by metagenomic sequencing. Each row represents data from a maternal subject (top) or cord plasma sample (bottom). The viral genera detected are indicated in the columns. A dark bar indicates the virus was detected in that subject.

**Figure 2:**
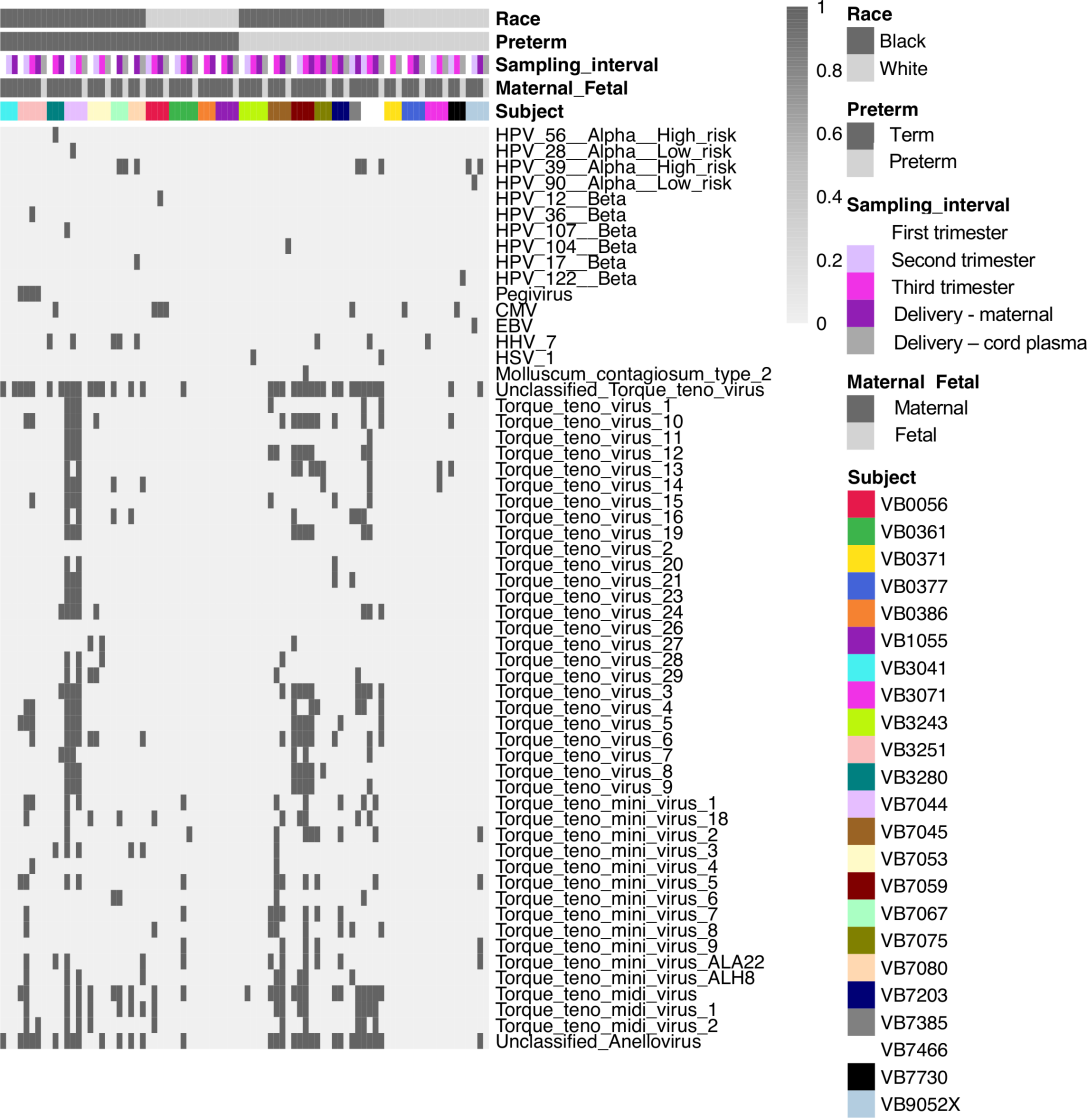
Viral types detected in maternal and cord plasma by metagenomic sequencing. Each column shows data from an individual sample from a subject. The top 5 rows indicate how samples are grouped based on race, preterm status, sampling interval (e.g. trimester of sampling or delivery), maternal or fetal sample, and subject. Each of the remaining rows represents data from an individual virus. A dark bar indicates the virus was detected in that sample.

**Figure 3:**
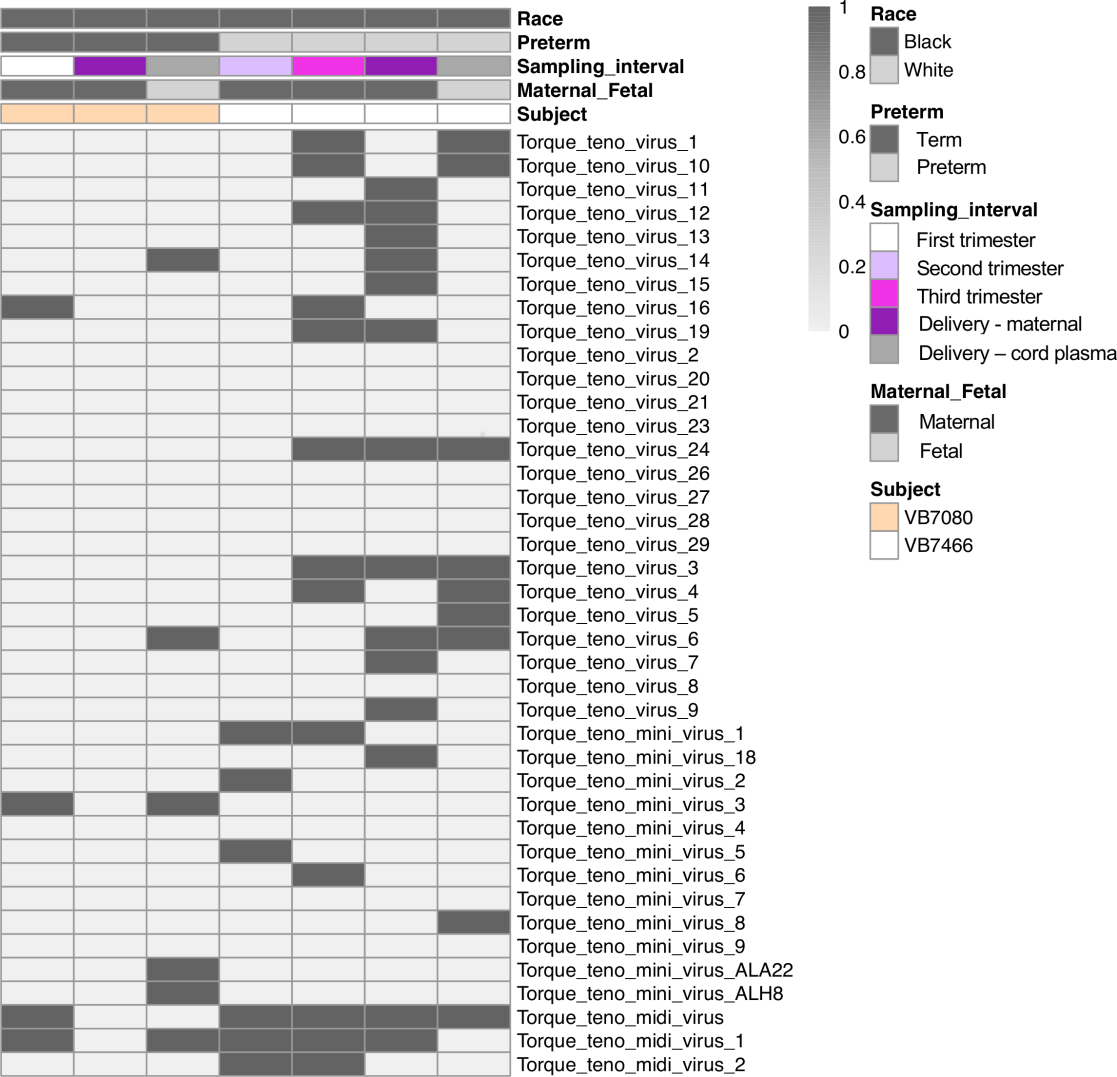
Anellovirus distribution in the two anellovirus-positive cord plasma samples and the corresponding maternal samples. Each column shows data from an individual sample from a subject. The top 5 rows indicate how samples are grouped based on race, preterm status, sampling interval (e.g. trimester of sampling or delivery), maternal or fetal sample, and subject. Each of the remaining rows represents data from an individual virus. A dark bar indicates the virus was detected in that sample.

To determine the composition and dynamics of the plasma virome during pregnancy we sequenced longitudinal samples from 23 pregnant patients, 14 Black and 9 White and cord plasma from their infants (**Table 1**). We detected nucleic acid from at least one virus from 87% (20/23) of the maternal subjects at some point during pregnancy (**Figure 1**). The viruses represented 11 genera from five families: *Herpesviridae, Poxviridae, Papillomaviridae, Anelloviridae*, and *Flaviviridae.* We detected nucleic acid from at least one virus in 33% (6/18) of the cord plasma samples (**Figure 1**). Viruses in cord plasma from the families *Herpesviridae, Papillomaviridae*, and *Anelloviridae.* Anelloviruses, which are known to be frequently detected in blood and other tissue, were the most common viruses detected in maternal samples (19/23). Many viral types were detected within the *Papillomaviridaae* and *Anelloviridae* (**Figure 2**). While nucleic acid from some viruses was detected transiently, others, including cytomegalovirus, pegivirus, HPV-39 and many anelloviruses were detected consistently over time in some patient samples (**Figure 2**). In the only two cord plasma samples that we detected anellovirus nucleic acid, both were from neonates born to maternal subjects who were also positive (**Table 2** and **Figure 3**). In one case, we detected nucleic acid from a mixture of anelloviruses, including the *Betatorquevirus* torque teno mini virus 3 and *Gammatorquevirus* torque teno midi virus 1 that were found in both cord plasma and the first trimester maternal sample. While no anellovirus sequences were detected in the maternal sample at delivery, it is possible that the viral nucleic acid was below the limit of detection. No second or third trimester samples were available for that patient. In the second case, in cord plasma we detected a mixture of *Alphatorquevirus* (including torque teno virus types 1, 3, 4, 6, 10, and 24)*, Betatorquevirus* (including torque teno mini virus 8), and *Gammatorquevirus* (including an unclassified torque teno midi virus type). That maternal subject was positive for a mixture of anelloviruses from the second trimester onward, including at delivery. No first trimester sample was available. While the torque teno virus 5 and torque teno midi virus 8 were only detected in the fetal samples, the other viruses were detected in at least one maternal sample (**Figure 3**). Cytomegalovirus (CMV) was detected in five maternal subjects (occurring in 1 antenatal sample in 4 subjects and antenatal and at delivery in 1 subject). It was also detected in a cord plasma sample obtained from the baby whose mother was positive antenatally and at delivery, although the infant did not develop a clinical CMV infection (**Table 2**). Herpes simplex virus 1 (HSV-1) was detected in one maternal subject and a one cord plasma sample, although those were not from matched maternal-neonatal pairs (**Table 2**).

**Table 1:** Characteristics of cohort.

Characteristic	Total cohort	ViroCap sequencing	Anellovirus PCR
Total number of subjects	63	23	63
Total number of samples	226	67	226
Trimester 1 (through week 13)	57	17	57
Trimester 2 (weeks 14-26)	53	13	53
Trimester 3 (weeks 27-40)	58	18	58
Delivery	58	19	58
Cord plasma	18	18	0
Maternal race			
Black	34	14	34
White	29	9	29
Term/Preterm			
Term	51	11	51
Preterm	12	12	12

**Table 2:** Viruses detected longitudinally by sequencing.

Patient	Race	Preterm	First Trimester	Second Trimester	Third Trimester	Delivery	Fetal cord plasma
**1**	Black	Term	Anellovirus	Anellovirus	NA*	Anellovirus	NA
**2**	Black	Term	HPV-36, Pegivirus, Anellovirus	Pegivirus, Anellovirus	Pegivirus, Anellovirus	Pegivirus, Anellovirus	None
**3**	Black	Term	Anellovirus	NA	Human herpesvirus 7, Anellovirus	HPV-56, Cytomegalovirus	NA
**4**	Black	Term	HPV-107, Anellovirus	HPV-28, Human herpesvirus 7, Anellovirus	Anellovirus	NA	None
**5**	Black	Term	Anellovirus	Anellovirus	Anellovirus	NA	None
**6**	Black	Term	Human herpesvirus 7, Anellovirus	NA	NA	Human herpesvirus 7, HPV-39, Anellovirus	HPV-39
**7**	Black	Term	Anellovirus	NA	NA	Human herpesvirus 7, HPV-39, HPV-17	Anellovirus
**8**	White	Term	NA	HPV-12, Cytomegalovirus	None	Cytomegalovirus, Anellovirus	Cytomegalovirus
**9**	White	Term	Anellovirus	None	None	Anellovirus	None
**10**	White	Term	None	NA	None	None	NA
**11**	White	Term	None	NA	None	None	None
**12**	Black	Preterm	Herpes simplexvirus 1	None	None	Anellovirus	None
**13**	Black	Preterm	Anellovirus	NA	Anellovirus	Anellovirus	HPV-104
**14**	Black	Preterm	Molluscum contagiosum virus type 2, Anellovirus	Anellovirus	Anellovirus	Anellovirus	NA
**15**	Black	Preterm	NA	NA	Human herpesvirus 7, Anellovirus	Anellovirus	None
**16**	Black	Preterm	NA	NA	Anellovirus	Anellovirus	None
**17**	Black	Preterm	NA	Cytomegalovirus, Anellovirus	NA	HPV-39, Anellovirus	NA
**18**	Black	Preterm	NA	Anellovirus	HPV-39, Anellovirus	Human herpesvirus 7, Anellovirus	Herpes simplex 1, HPV-39, Anellovirus
**19**	White	Preterm	None	NA	None	None	None
**20**	White	Preterm	None	NA	Cytomegalovirus	None	None
**21**	White	Preterm	Human herpesvirus 7	None	Anellovirus	NA	None
**22**	White	Preterm	NA	Anellovirus	Cytomegalovirus	NA	HPV-122
**23**	White	Preterm	HPV-39, Anellovirus	HPV-39	NA	Epstein Barr Virus, HPV-90	None

*”NA” indicates there was no sample tested at that timepoint. “None” indicates the sample was tested, but no virus was detected. Anellovirus subtypes are shown in **Figure 2**.

**Figure 4:**
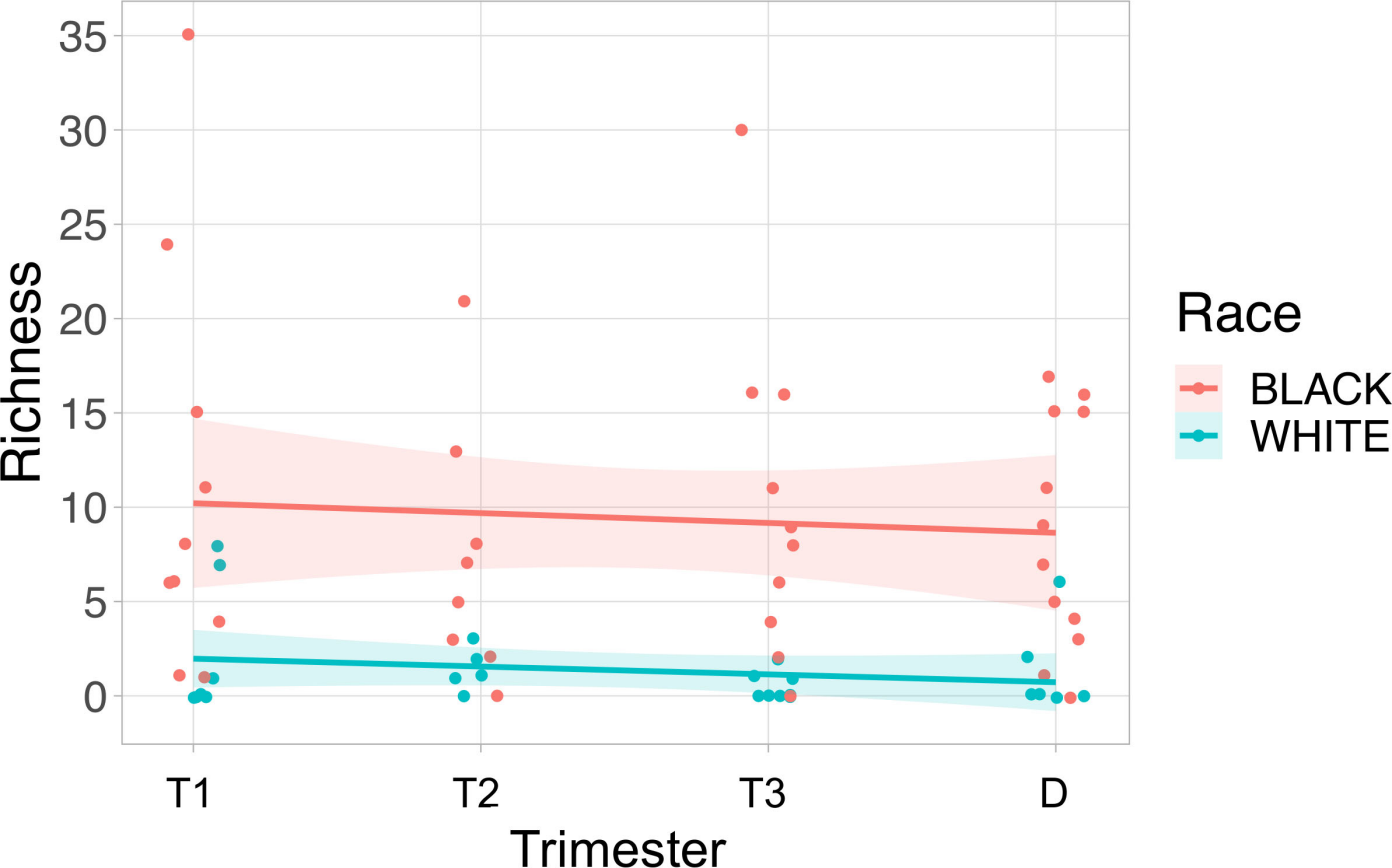
Viral richness in maternal plasma is higher in Black subjects than White subjects. Viral richness (the number of different viruses detected) is plotted on the y-axis, and the time point the sample was collected was plotted on the x-axis. The fitted line is plotted with the 95% confidence interval shaded. Black subjects are plotted in pink, and White subjects are in blue. T1, Trimester 1; T2, Trimester 2; T3, Trimester 3; D, Delivery.

We evaluated viral richness (the number of different viruses detected) throughout pregnancy. The maximum number of distinct viral types detected in a single sample was 35. Using a linear mixed effects model, we found that Black race was associated with higher viral richness in the maternal samples (P=0.003) (**Figure 4**). We did not detect associations between viral richness and PTB (delivery less than 37 weeks, n=12) or the gestational age of sampling during pregnancy.

### Anellovirus nucleic acid positivity and genome copy numbers

**Figure 5:**
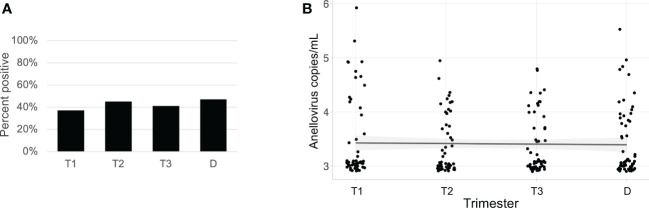
Anellovirus positivity and load are consistent throughout pregnancy in the full data set. In **(A)**, the percentage of maternal samples positive by PCR assay (y-axis) is shown for each time point (x-axis). In **(B)**, the anellovirus copy number (load) in copies per mL (y-axis) is shown for each time point (x-axis), and the fitted line is plotted with the 95% confidence interval shaded. T1, Trimester 1; T2, Trimester 2; T3, Trimester 3; D, Delivery.

**Figure 6:**
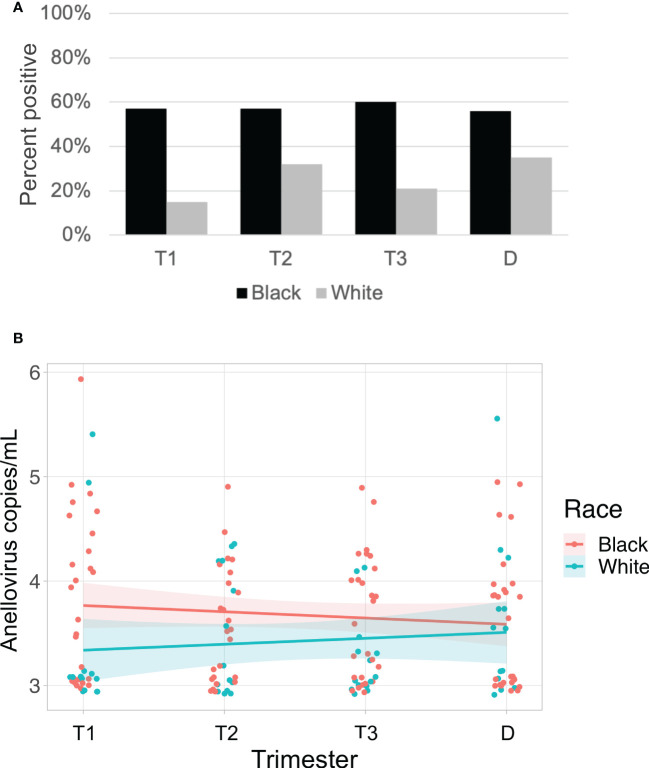
Anellovirus positivity is higher in Black subjects than White subjects. In **(A)**, the percentage of maternal samples positive by PCR assay (y-axis) is shown for each time point (x-axis). Results from Black subjects are indicated by black bars; White subjects are indicated by gray bars. In **(B)**, only subjects who were positive for anellovirus in at least one of their samples are included. The anellovirus genome copies per mL (y-axis) is shown for each time point (x-axis), and the fitted line is plotted with the 95% confidence interval shaded. Black subjects are plotted in pink, and White subjects are in blue. Black and White groups differed by positivity (P<0.001) but not by viral load (P=0.1) by generalized linear mixed effects model. T1, Trimester 1; T2, Trimester 2; T3, Trimester 3; D, Delivery.

**Figure 7:**
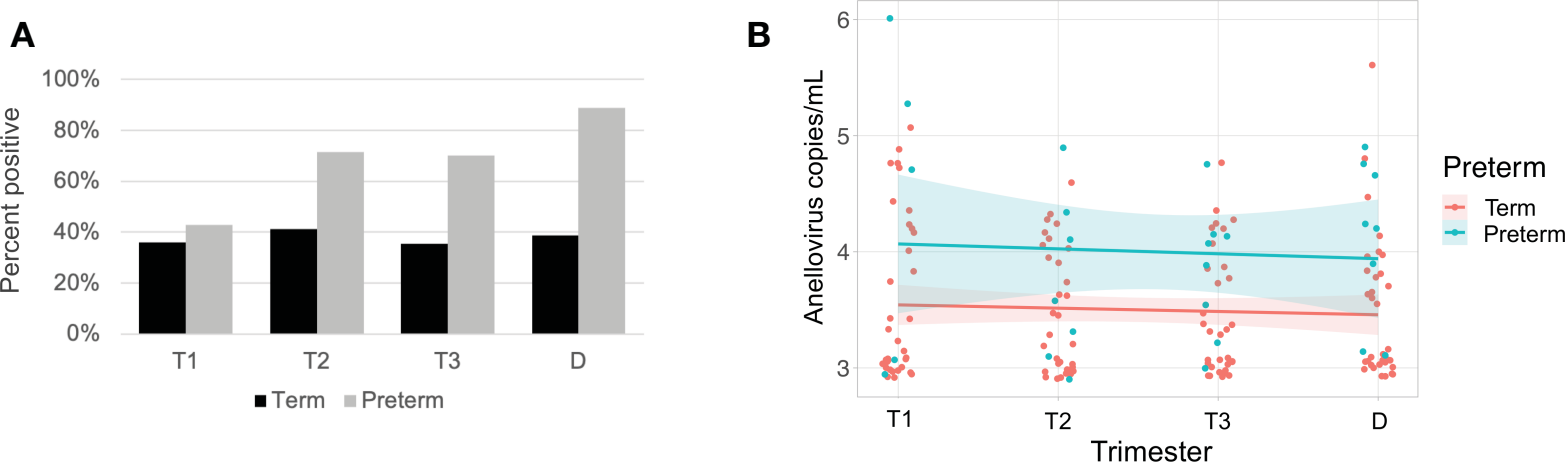
Anellovirus positivity and load are higher in subjects that deliver preterm compared with subjects that deliver at term. In **(A)**, the percentage of maternal samples positive by PCR assay (y-axis) is shown for each time point (x-axis). Results from term subjects are indicated by black bars; preterm subjects are indicated by gray bars. In **(B)**, only subjects who were positive for anellovirus in at least one of their samples are included. The anellovirus copies per mL (y-axis) is shown for each time point (x-axis), and the fitted line is plotted with the 95% confidence interval shaded. Term subjects are plotted in pink, and preterm subjects are in blue. Term and preterm groups differed by both positivity (P<0.01) and viral load (P=0.003) by generalized linear mixed effects model. T1, Trimester 1; T2, Trimester 2; T3, Trimester 3; D, Delivery.

Anelloviruses were the most common viruses in the samples, so we used quantitative PCR in an expanded cohort (N=63) to assay anellovirus genome copy numbers in plasma samples collected during each trimester and at delivery to determine whether there were dynamic changes associated with progression through pregnancy. 54% (N=34) were Black and 46% (N=29) were White; 19% (N=12) delivered preterm (**Table 1**). There was no change by trimester in anellovirus positivity (**Figure 5A**) or genome copy number (**Figure 5B**) in the plasma. However, we found that there was higher anellovirus positivity (**Figure 6A**, P<0.001) and a trend toward higher genome copy numbers (**Figure 6B**, P=0.1) associated with Black race across all trimesters in pregnancy. Using the quantitative PCR data, we also evaluated associations between anellovirus and PTB. We found that higher anellovirus positivity was associated with PTB (**Figure 7A**, P<0.01). Higher anellovirus genome copy number was also associated with PTB (**Figure 7B**, P=0.003).

## Discussion

Our analysis of the plasma virome showed anelloviruses are the most prevalent and abundant viruses in pregnant patients. This is expected since this viruses is consistently the predominant virus in the blood starting during childhood (McElvania TeKippe et al., 2012; Wylie et al., 2012; Moustafa et al., 2017; Sloan et al., 2022). We found the rate of anellovirus positivity and viral load was stable throughout pregnancy, despite the physiologic immune changes associated with pregnancy. While other studies have evaluated single time points (Shah et al., 2020; Sloan et al., 2022), our longitudinal data demonstrate the absence of a specific pattern of change in virome composition, anellovirus positivity or anellovirus load associated with trimester of pregnancy. Shah, et al. posited that anellovirus positivity could be a signal for parturition. Our data do not support that conclusion, as samples from term deliveries did not increase in positivity or load at the time of delivery compared to samples obtained during the antenatal course.

Our study did find an association between anellovirus positivity and copy number with PTB, which is consistent with other complementary studies. In one study, positivity for any anellovirus was associated with spontaneous preterm labor (Shah et al., 2020); although we considered all preterm birth regardless of subtype, our study also supports this link. In a second study, only preterm pregnancies were evaluated, and no difference in anellovirus positivity or copy number was observed between various types of spontaneous and indicated PTB, which supports the validity of grouping PTB for our analysis (Sloan et al., 2022). We have previously reported higher vaginal viral richness in vaginal swabs from pregnant patients who had PTB compared with term births (Wylie et al., 2018b), although no single viral family was associated with PTB. In contrast, in the present analysis of the maternal blood virome, we found that anelloviruses were associated with PTB.

Our observation that viral diversity and anellovirus positivity were higher in Black patients compared to White patients reinforces the importance of accounting for population characteristics in studies of the virome and microbiome. This view is consistent with our findings from a study of the virome in vaginal samples from pregnant patients, where viral diversity was also higher in Black patients compared to White patients (Wylie et al., 2018b). These findings raise the possibility that the viral differences detected in our cohort are not a consequence of race, but instead are a consequence of other unmeasured exposures experienced differently by different racial and ethnic groups (Bailey et al., 2017; Mateo and Williams, 2021; Johnson and Louis, 2022). Additionally, since we have not evaluated paired vaginal and serum samples from the same patient it is not known whether higher viral diversity in the vagina correlates with higher diversity in blood or possibly even nasal, skin, or stool samples. It is not clear whether higher diversity of viruses in the blood and vagina could share a common cause.

In addition to *Anelloviridae*, nucleic acid from viruses from four other families were detected in maternal plasma, with at least one virus present in approximately 87% of individuals and a maximum of 35 viruses in one individual. In contrast, only about one third of cord plasma specimens had any virus detected, which included very few anelloviruses, despite their prevalence in maternal samples. These data are consistent with a previous study that used PCR to evaluate specific viruses (Sloan et al., 2022). Interestingly, we found examples of the same papillomavirus (HPV type 39, a potentially oncogenic alphapapillomavirus) in multiple samples from patients collected over time and passed into the cord plasma. This suggests detection of HPV DNA in the plasma may not always be transient or explained by potential contamination from the skin during blood collection, as has been posited. It would be interesting in future studies to correlate components of the virome in peripheral sites (vagina, nose, stool) with viruses detected in the blood from pregnant women to determine whether peripheral sites may be a source for circulating viral nucleic acid in asymptomatic patients. Herpesvirus infections are very common, thus it was not surprising that we detected herpesvirus nucleic acid in the blood plasma from several of the patients. As the cell-free plasma was tested, these results suggest that we detected viruses that had reactivated and were circulating. We employed the highly sensitive ViroCap enrichment method with metagenomic sequencing, which allowed us to comprehensively assess vertebrate viruses in these samples. The results from this approach suggest that we are not missing rare or unusual viruses in our analysis of cord plasma and suggest there is very low transplacental passage of viruses to the infant. However, it is also possible that viruses in cord plasma could be below the detection limits of the technique.

There are several strengths of this study of the maternal virome. First, longitudinal sampling during pregnancy allows us to evaluate dynamic changes in virome characteristics over time. Second, a racially diverse cohort was evaluated, revealing significant differences in virome characteristics between Black and White patients. These findings raise important questions regarding exposures and experiences of populations to be explored in future studies. Finally, we employed a much broader analysis of the plasma virome during pregnancy, using the ViroCap enrichment method to improve virus detection with sequencing, than prior studies of viral infections which either studied only the classic and some emerging viruses (Chudnovets et al., 2020; Sloan et al., 2022; Yu et al., 2022) or used metagenomic sequencing of a small number of samples (Shah et al., 2020). A weakness of our study is the relatively small number of PTB patients. Finally, although we did not differentiate between anellovirus subtypes (King et al., 2012) or genogroups (Maggi et al., 2005) in our PCR assay unlike other studies (Shah et al., 2020; Sloan et al., 2022), our assay of all anelloviruses still revealed associations with race and PTB. Future studies will be aimed at expanding the study to address these limitations and explore the underlying causes for associations between the virome with race and PTB.

## Data availability statement

The data presented in the study are deposited in the Sequence Read Archive, accession number PRJNA892083.

## Ethics statement

The studies involving human participants were reviewed and approved by The Human Research Protection Office at Washington University School of Medicine. The patients/participants provided their written informed consent to participate in this study.

## Author contributions

MS and KW conceived of and designed the study, analyzed data, and interpreted results. AB, BH, and AR contributed to data generation, analysis, and/or interpretation. AB and AR contributed to a review of the literature. AB and KW wrote the first draft of the manuscript. All authors contributed to the article and approved the submitted version.
